# Melting temperature signatures reveal occasional misclassification of low-level isoniazid resistance by Xpert MTB/XDR

**DOI:** 10.1128/spectrum.00713-26

**Published:** 2026-07-24

**Authors:** Faridath Massou, Jelle Keysers, Hervé Kpoton, Bouke C. de Jong, Philip Supply, Leen Rigouts, Dissou Affolabi

**Affiliations:** 1 Supranational Reference Laboratory of Tuberculosis, Cotonou, Bénin; 2 Mycobacteriology Unit, Department of Biomedical Sciences, Institute of Tropical Medicine 37463 , Antwerp, Belgium; 3 Faculty of Pharmaceutical, Biomedical and Veterinary Sciences, University of Antwerp 26660, Antwerp, Belgium; 4 Univ. Lille, CNRS, Inserm, CHU Lille, Institut Pasteur de Lille, U1019–UMR 9017, Center for Infection and Immunity of Lille, Lille, France; Ascension St John Hospital, Detroit, Michigan, USA

**Keywords:** *fabG1*/*inhA*, melting temperature analysis, isoniazid resistance, Xpert MTB/XDR

## LETTER

Isoniazid (INH) remains one of the most potent and affordable anti-tuberculosis drugs. Although standard WHO-recommended regimens for rifampicin-resistant TB are currently bedaquiline-based (1) and do not include INH, accurate identification of INH resistance may remain relevant in individualized treatment contexts, such as linezolid toxicity (2) or regimen optimization, particularly given approximately 22% (3) of rifampicin-resistant patients remain susceptible to INH. Accurate identification of INH resistance is also essential in INH mono-resistant TB patients, managed using modified rifampicin-based regimens (1). Mutations in *katG* and *inhA* confer different resistance levels, with *katG_S315T* generally associated with higher minimal inhibitory concentrations (MICs), whereas *fabG1-inhA* promoter variants are mostly associated with lower MICs increases, although variability exists (4).

The GeneXpert MTB/XDR assay (Cepheid, USA) targets *katG*, the *fabG1-inhA* promoter, and other regions (*fabG1*, *oxyR-ahpC*) using sloppy molecular beacons and melt curve analysis to detect INH resistance (5). While the cartridge output reports INH resistance based on the target regions (Fig. 2A and B), raw melting temperature (Tm) profiles may help infer specific mutations and/or generate minimal molecular epidemiological data.

We analyzed 872 clinical *Mycobacterium tuberculosis* complex samples with paired Xpert MTB/XDR and Deeplex Myc-TB (GenoScreen, France) results (6), supplemented by whole-genome sequencing for Deeplex Myc-TB out-of-target variants. For each probe region, Tm values were extracted and compared with sequencing results.

**Fig 1:**
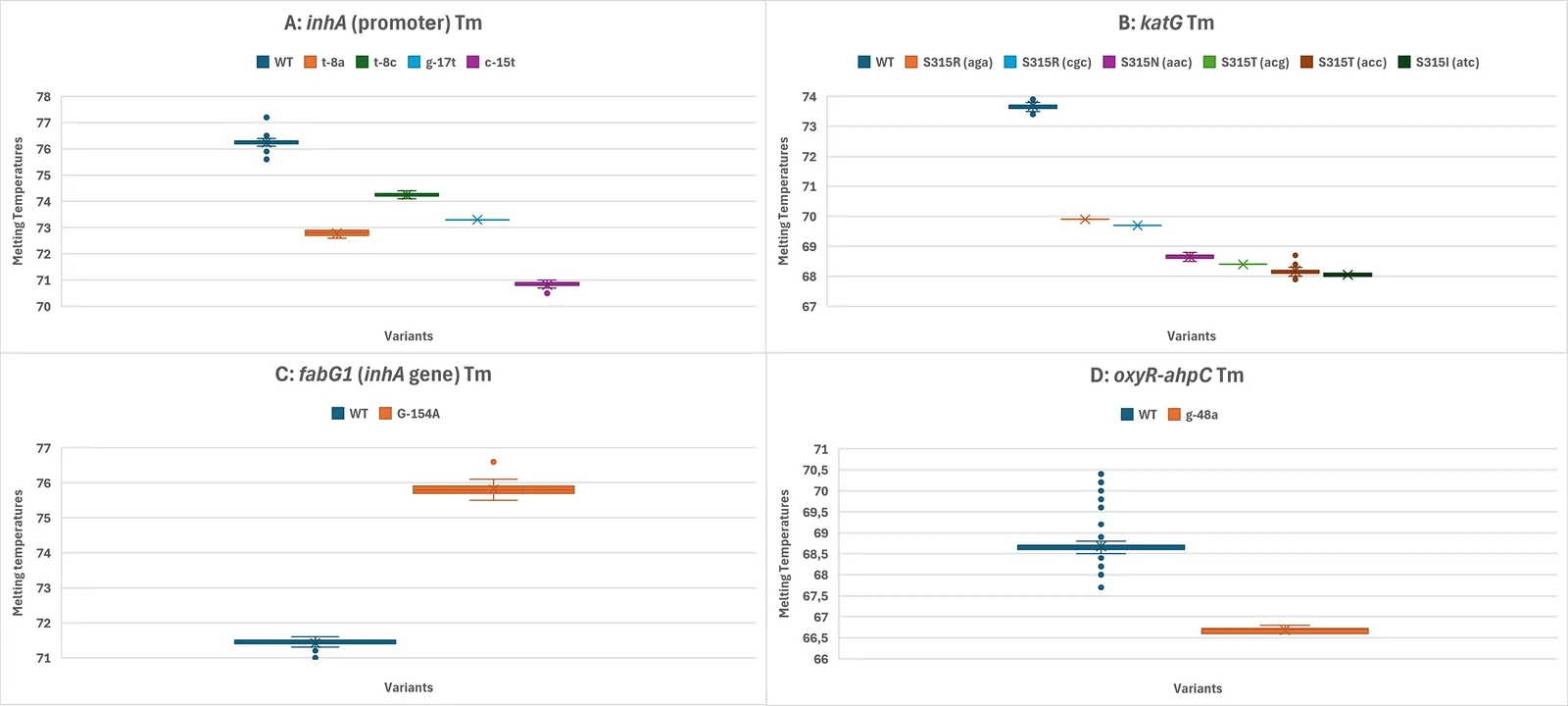
Distribution of melting temperatures (Tm) for wild-type and mutant variants across INH resistance-associated genes. Each dot represents the Tm value obtained for an individual sample. Horizontal bars indicate the mean Tm for either the WT or a specific variant, and vertical bars represent the standard deviation. (**A**) *inhA* promoter, (**B**) *katG* gene, (**C**) *fabG1* (*inhA* gene), and (**D**) *ahpC* promoter.

**Fig 2:**
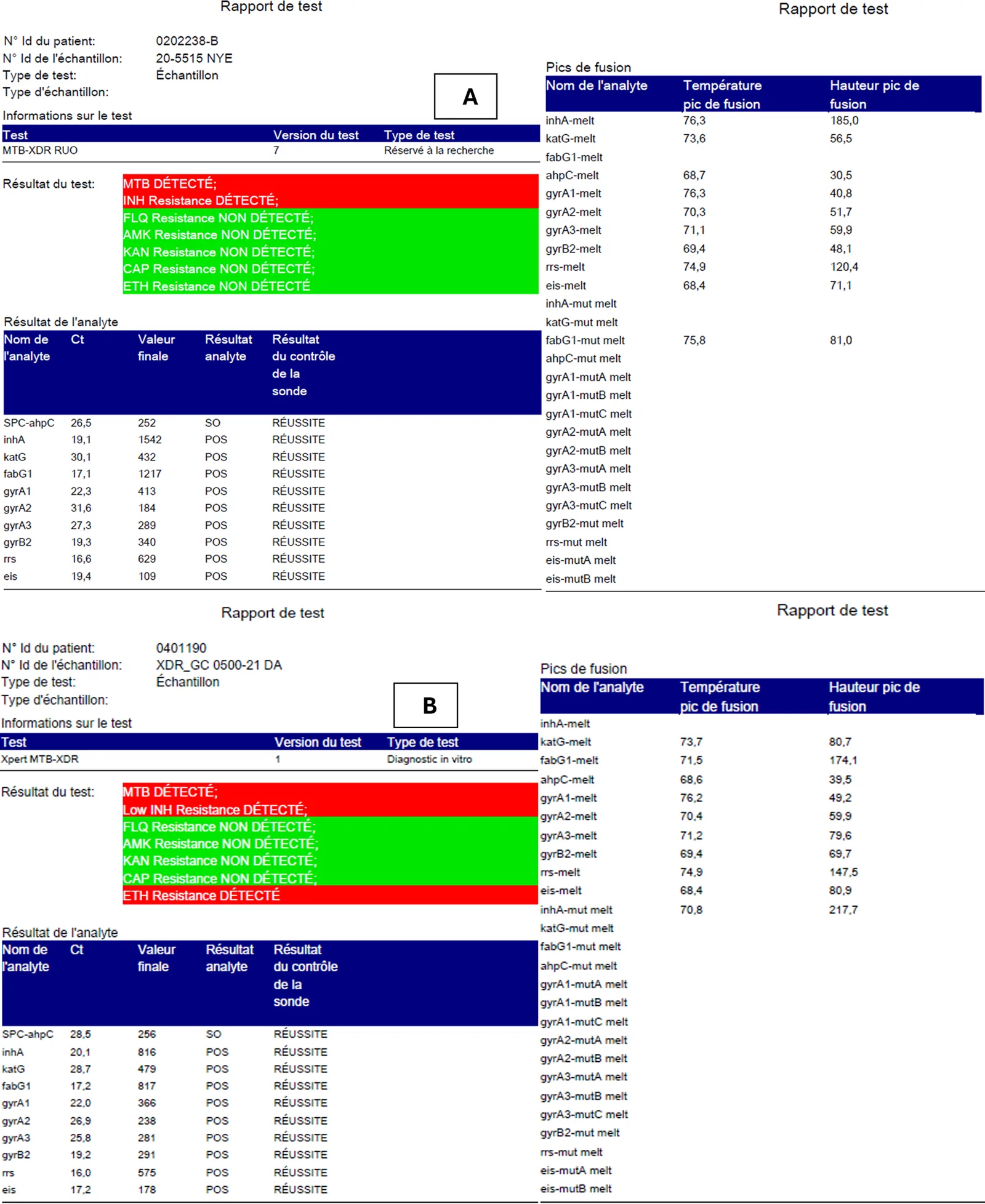
Panels** A **(INH resistance detected) and **B** (low INH resistance detected) illustrate RUO and IVD reporting formats obtained using the same Xpert MTB/XDR assay. Screenshots correspond to original instrument-generated reports (French interface). Translations: N° ID du patient = patient ID; N° ID de l’échantillon = sample ID; Type de test = test type; Résultat du test = test result; Résultat de l’analyte = analysis result; Nom de l’analyte = analyte name; Température pic de fusion = melting peak temperature; Hauteur pic de fusion = melting peak height; Résistance détecté = resistance detected; Résistance non détecté = resistance not detected; Low INH Résistance détecté = low INH resistance detected.

Wild-type (WT) and mutant alleles were generally well discriminated across loci (5), with WT consistently showing higher Tm values than mutants (Table 1, Fig. 1) except for *inhA*_c.-154G>A (formerly *fabG1*_p.L203L). This mutation occurs alone in 3.7% of INH-resistant isolates in the WHO catalog v2 where it is classified as conferring low-level INH resistance and cross-resistance to ethionamide (ETH) (7). Although missed by Deeplex Myc-TB and line probe assays (8, 9), it was reported by Xpert MTB/XDR as “resistance detected,” thereby implicitly categorizing it as high-level resistance (Fig. 2A and B) without indicating ETH resistance. All culture-positive samples carrying this variant were INH-resistant by phenotypic drug-susceptibility testing (pDST, Löwenstein-Jensen; proportion method; 0.2 µg/mL [10]). As neither INH-MICs nor ETH-pDST were performed, the level of resistance conferred by this mutation relied on the WHO catalogue (7), highlighting the potential need for future MIC-based studies to confirm the resistance level.

**TABLE 1:** Xpert MTB/XDR melting temperatures vs mutations identified by Deeplex Myc-TB^*a*^^,^^*f*^^,^^*g*^

Deeplex Myc-TB	Number	WHO cat	Tm mean	Tm median	Tm mode	Tm min	Tm max
A: *inhA* promoter							
WT	734	NA	76.24	76.20	76.20	75.60	77.20
t-8a	15	Cat 2	72.78	72.80	72.80	72.60	72.90
t-8c	33	Cat 2	74.25	74.20	74.20	74.10	74.40
c-15t	87	Cat 1	70.81	70.80	70.80	70.50	71.0
g-17t	2	Cat 2	73.30	73.30	73.30	73.30	73.30
B: *katG*							
WT	521	NA	73.69	73.70	73.70	73.40	73.90
S315I (atc)^*b*^	2	Cat 2	68.05	68.05	NA	68.0	68.10
S315N (aac)^*b*^	17	Cat 1	68.66	68.70	68.70	68.50	68.80
S315T (acc)^*b*^	316	Cat 1	68.18	68.20	68.20	67.90	68.70
S315T (acg)^*b*^	1	Cat 1	68.40	68.40	NA	68.40	68.40
S315R (aga)^*b*^	3	Cat 1	69.90	69.90	69.90	69.90	69.90
S315R (cgc)^*b*^	1	Cat 1	69.70	69.70	NA	69.70	69.70
C: *fabG1* (*inhA* gene)							
WT	803	NA	71.42	71.40	71.40	71.0	71.60
(g-154a)^c^	58	Cat 1	75.81	75.80	75.70	75.5	76.60
D: *oxyR-ahpC*							
WT	847	NA	68.67	68.70	68.70	67.7	70.4
g-48a^*d*^	6	Cat 3	66.68	66.70	66.70	66.6	66.8
c-57^*d*^	1	Cat 3	66.00	66.00	NA	66	66.0
t-75g^*e*^	1	Cat 3	67.60	67.60	NA	67.6	67.6
g-90a^*e*^	1	Cat 3	66.30	66.30	NA	66.3	66.3

^*a*^ WT, wild type; Tm, melting temperatures, uppercase letters, amino acid; lowercase letters, nucleotides; NA, not applicable.

^*b*^ Observed or potential overlap (if more tested).

^*c*^ Mutations out-of-target by Deeplex Myc-TB but detected by Xpert MTB/XDR.

^*d*^ WHO cat 3 upstream *ahpC* variants classified as resistant by Deeplex Myc-TB, based on known compensatory effect of isoniazid resistance fitness cost.

^*e*^ Mutations classified INH R by Xpert MTB/XDR but uncharacterized by Deeplex Myc-TB.

^*f*^ Numbers represent samples per target region; as some samples carried mutations in multiple genes, totals across loci do not sum to the overall sample size.

^*g*^ Evidence for compensatory effects was reported for all listed upstream *ahpC* mutations in reference 11.

For *katG* variants, Tm distributions of S315T, S315N, and S315I grouped separately from that of S315R; however, interpretation of mutation-specific Tm ranges remains limited for variants with low numbers of samples. In contrast, *inhA* promoter mutations (c-15t, t-8a/c, g-17t) displayed well-separated Tm values.

*ahpC* upstream variants were less frequent, limiting detailed analysis. These compensatory mutations (11) and surrogate resistance markers of INH resistance, often co-occurring with *katG* mutations, can trigger INH-resistant calls by Xpert MTB/XDR despite not being classified as resistance-associated in the WHO catalog v2 themselves (12).

In conclusion, Xpert MTB/XDR provides consistent Tm signatures for INH resistance detection but misclassifies the *fabG1*_p.L203L variant as high-level rather than low-level resistance and does not report its cross-association with ETH resistance in contrast to the WHO catalogue. Explicit reporting of both low-level and high-level resistance would improve clarity for end users and enhance the clinical value of the assay.

## SUPPLEMENTAL MATERIAL

Data S1 spectrum.00713-26-s0001.xlsx Database with all the test results.

ASM does not own the copyrights to Supplemental Material that may be linked to, or accessed through, an article. The authors have granted ASM a non-exclusive, world-wide license to publish the Supplemental Material files. Please contact the corresponding author directly for reuse.
